# Supplementary material for: Food consumption score and predictors among pregnant women attending antenatal care services in health centers of Addis Ababa, Ethiopia: Using ordinal logistic regression model
Source: PLoS One. 2024 Jun 26;19(6):e0306169. doi: 10.1371/journal.pone.0306169 (PMC11207176; doi:10.1371/journal.pone.0306169)
Supplement: Attachment — Submitted filename: peer review coments.docx [file pone.0306169.s002.docx]

I have read this submission, and it addresses an important topic. However, I have questions about the method, independent variables, result, and conclusion in addition to the major issue regarding the food consumption score.

**Abstract**: What does it mean that understanding the food consumption pattern can prevent pregnancy related problems? Since the abstract gives us all the information regarding the document, it is expected to mention important things. So, how do you measure the food consumption score?

**Background/introduction**: needs to be rewritten properly and improve spelling and grammatical errors e.g vitamin---should be vitamins, ………. And do not capitalize words that are not the first in a sentence I have concerns about the justification/rationale of the study since there are a number of studies published in this area

**Methods**: During data collection were there pregnant women who were seriously ill Sampling technique multistage why? Clearly describe practically how do you get your study subjects Data collection tool: after the pre-test you mentioned that you excluded residence why did you consider it at first because the study area was urban i.e. Addis Ababa? And what measure have you taken after pre-test for food consumption score? What type of variable does your questioner contain? How do you measure attitude I think you mention in the result section? Major issue: for your outcome variable (FCS) rewrite how you measure it You used reference to categorize FCS for households why do not use for pregnant? i.e. consists category of 0-28, 28.5-42, >42 As we all know in our country, micronutrient deficiency during pregnancy are common; however, FCS has been validated against quantity of caloric intake, it has not been validated against adequacy of macronutrients or micronutrients so, why don’t you supplement with other dietary methods? To assess economic status you mentioned the wealth index was used even if you did not mention how you conducted and how you made the analysis, wealth index is most commonly applied to rural communites since they do not know their exact economic source or monthly income and have agricultural products but your study setting was urban?so, why you conduct wealth index?

**Analysis:** the statistical analysis is not adequate Lack of necessary data How do you analyse attitude?

**Result:** rewrite the result systematically, and the tables need to be summarized Some of your variables of categories have a too small frequency e.g. maternal education which accounts less than 10%

**Discussion**: correct more than half is 51.2% more than half? Your discussion was not written coherent manner, and it does not keep the writing flow, you just once mentioned local and then abroad studies  And while you justify it was mentioned that the study provided greater evidence on dietary quality how? You utilized only FCS how do you measure dietary quality? What measures did you take to manage recall bias?

**Conclusion:** rewrite the conclusion that is out of your result

**Reference** : there are too old reference better to use latest sources
