# Supplementary material for: Food consumption score and predictors among pregnant women attending antenatal care services in health centers of Addis Ababa, Ethiopia: Using ordinal logistic regression model
Source: PLoS One. 2024 Jun 26;19(6):e0306169. doi: 10.1371/journal.pone.0306169 (PMC11207176; doi:10.1371/journal.pone.0306169)
Supplement: Attachment — Submitted filename: Response to reviewers .docx [file pone.0306169.s003.docx]

**Date: May 9: 2023**

To: Plos One, Editorial Office

Subject: Submitting a revised version of manuscript and point by point response

Manuscript ID: PONE-D-23-02910

Title: Food consumption score and predictors among pregnant women attending antenatal care services in health centers of Addis Ababa, Ethiopia: using ordinal logistic regression model

We would like to thank the editor and the reviewers for sharing their view and experience on the manuscript. We have found the comments are very important, which will improve the quality of our manuscript. The point-by-point responses for each of the comments and the revised manuscript are provided in the attached documents.

Regards,

Authors

**Point -by-point response**

**Editorial requests:**

| **Editors Report** | **Authors’ response** |
| --- | --- |
| Results: Why were the proportions of FCS out of the CI? | Dear Editor,  Thank you for your comments, this was occurred due to a typological error. Now we have corrected these errors. For example, on the abstract section it was previously written as (4.80-5.44), but now it has been corrected this way (48.0%-54.4%). Therefore, it has been corrected the CI including FCS. The amended CI can be found on line # 47, #48, #217, # 218, #219 and # 251. |
| Why did you use mean or +SD? | Dear editor,  We used mean or +SD for the continuous variables which did not have extreme values. For example, age on line #186. |
| How did you determine the variables for the wealth index to be computed for further analyses? | We used principal component to compute after checking all the assumptions such as sampling adequacy and Kaiser-Meyer-Olkin (KMO) measures. |
| How many questions were used to assess the attitude? | To assess the attitude of mothers towards the consumption variety of diets we used 4 questions. It was 4 item Likert scale questions with responses ranging from strongly agree to strongly disagree.  The following has been written in the modified manuscript from line # 158 to #162.  ‘The attitude of the study participants towards the consumption diversified diet was measured using a 4 item Likert-scale questions, the response ranges from strongly disagree to strongly agree. It was considered favourable attitude when respondents score above the median. The internal consistency of the questionnaire was checked using Cronbach’s alpha (0.78). ’ |

**Point by point response to reviewers**

**Reviewer 1**

| **Reviewers report** | **Authors’ response** |
| --- | --- |
|  | Dear Reviewer,  We would like to offer our heartfelt gratitude for being the reviewer of our manuscript, we optimist that your comments will augment the quality our manuscript. Therefore, the following are point by point response for the raised questions and comments. |
| **Abstract**:  What does it mean that understanding the food consumption pattern can prevent pregnancy related problems? | Dear Reviewer,  What we wanted to address there is understanding food consumption pattern by assessing the magnitude and associated factors will help in preventing pregnancy related problems that might be due decreased food consumption score.  By assessing the food consumption score of a pregnant women one can tell the calorie adequacy of foods that a pregnant woman consumes. Besides, through this kind of study we can identify who is vulnerable, and we can also determine factors linked to low level of food consumption score. |
| Since the abstract gives us all the information regarding the document, it is expected to mention important things. So, how do you measure the food consumption score? | Dear Reviewer,  Thank you for your comments now we have incorporated the following statement on the methods section of the abstract- page 2 line #39-#42.  ‘Food consumption score was assessed after collecting data on frequency eight food groups consumed over the previous seven days, which were weighted according to their relative nutritional value.’ |
| **Background/introduction**: needs to be rewritten properly and improve spelling and grammatical errors e.g vitamin---should be vitamins, ………. And do not capitalize words that are not the first in a sentence | Dear Reviewer,  Thank you for your comments. We have made amendments based on the forwarded comment.  For example, the word ‘vitamin’ has been edited as vitamins. The capitalized letters which were not in the beginning of the sentence have also been in small letters. |
| I have concerns about the justification/rationale of the study since there are a number of studies published in this area | Dear Reviewer,  We do share your concerns, our justification behind the limited information on this topic in the light of FCS. We do know that FCS is a composite variable constructed in the following criteria: food frequency, diet diversity and relative nutritional value of each food item.  Of course, we did not deny the existence of handful of studies related to our topic. For example, in Ethiopia there were two studies one was conducted in Eastern Ethiopia while the other was conducted in Northwest Ethiopia (Reference #11 and #12). However, the two studies did not use ordinal logistic regression to ascertain associated factors.  In the two former studies, the outcome variable was considered as binary (acceptable and unacceptable food consumption score). However, food consumption score categorized into three categories: poor, borderline and acceptable FCS. Therefore, ordinal logistic regression is more precise than binary logistic regression to identify associated factors.  From different studies, we understand that ordinal logistic is better predictor of FCS than formerly used model, binary logistic regression. Unlike binary logistic regression, ordinal logistic considers the ordered nature of the outcome variable.  Besides, there is a limited information on FCS among pregnant mothers residing in big cities of Ethiopia as others studies are from rural and semi urban areas. |
| **Methods**: During data collection were there pregnant women who were seriously ill | Yes, there were pregnant mothers who were seriously ill during the data collection period. We have encountered 4 mothers who were seriously ill during the data collection period. These mothers who were seriously ill during the data collection period were excluded from the study (line # 125) |
| Sampling technique multistage why? Clearly describe practically how do you get your study subjects | Dear reviewer,  The reason for us to use a multi-stage sampling technique is that to get the study subjects we had go through different stages.  From line # 124 to # 132 the following statement has been written:  ‘Out of the nine sub-cities in Addis Ababa, four sub cities were selected randomly (30%) In the selected sub cities there were 28 health centres. First, nine health centres were selected randomly using a lottery method. Then, the required sample size was proportionated to the selected health centres, and every three (k≈ 2478/999) pregnant woman who was coming for ANC follow up was recruited.’  The selected health centers were: Woreda 01 HC, Alembank HC, Wereda 03 HC, Teklehaymanot HC, Beletshachew HC, Kality HC, Gelan HC, Kilinto HC and Abnet HC. These health centers are scattered over Addis Ababa.  Finally, after selecting these health centers the study subjects were selected using systematic random sampling-then every three pregnant woman coming for ANC follow up was selected, the first was selected randomly from the three using a lottery method. There, two was randomly selected, then we selected the 2^nd^, the 5^th,^ 8^th^ and it goes on until we find the final sample. Selected study subjects were included when they came for ANC services. |
| Data collection tool: after the pre-test you mentioned that you excluded residence why did you consider it at first because the study area was urban i.e. Addis Ababa? | Dear Reviewer,  As we have adapted the questionnaire from other studies that included rural areas, the question residency was considered as a variable. However, during the pretest we did not find a study participant who is from a rural area. |
| And what measure have you taken after pre-test for food consumption score? | Dear reviewer,  After the pre-test we have added some foods in some of the food groups. For example, ‘Chiko’ and ‘Chechebsa’ were added under main staples (cereals) category; and under the vegetables category, Fossolia was included after the pertest. From the cereal section, ‘Bokolo eshet’ which was not common and inaccessible during the study period.  Besides, to support the above explanation- we have now included the following statement from line # 141to # 143 'On the food frequency questionnaire, necessary modification was made by including foods that were not previously included.’  We have also added the following statement from line #150 to # 152  ‘In the questionnaire, there were 70 food items there were commonly consumed in the study area., |
| What type of variable does your questioner contain? | Dear reviewer,  The questionnaire contained questions which assess the sociodemographic socio-demographic data, dietary habits, attitude obstetric history, and food consumption score of the study participants (Line #134 to 136#). For example, age, marital, family size, husband education, husband occupation, educational status, occupation and wealth status were among the variable that were included under questionnaire that used to asess sociodemographic characteristics of the participants. |
| How do you measure attitude I think you mention in the result section? | Dear Reviewer,  Yes, you are right we have mentioned it in the result section. Now in the modified manuscript we have also included a statement that explain how we measured attitude from line # 158 to # 162:  ‘The attitude of the study participants towards the consumption diversified diet was measured using a 4 item Likert-scale questions, the response ranges from strongly disagree to strongly agree. It was considered favorable attitude when respondents score above the median. The internal consistency of the questionnaire was checked using Cronbach’s alpha (0.78).’ |
| Major issue: for your outcome variable (FCS) rewrite how you measure it You used reference to categorize FCS for households why do not use for pregnant? i.e. consists category of 0-28, 28.5-42, >42 | Dear Reviewer,  We appreciate the concerns. For the best of our knowledge there were two studies which assessed FCS among pregnant women in Ethiopia. The first study was conducted in Northwest Ethiopia at Shegawa Motta Hospital (reference #11), in this study WFP classification of FCS was used which is congruent to our study. The other study which was conducted in rural Eastern Ethiopia Harerege district (reference # 12), a different definition of FCS was seen - of 0-28, 28.5-42, >42.  As it has been indicated by the WFP report, the later threshold is used when there is a higher consumption of oil and sugar on daily basis.  However, neither of the study have tried to validate the tool among pregnant women in Ethiopia.  In cognizance of this fact, we opted to use the standardized classification that is outlined by WFP, which also used by an institution-based study in Ethiopia (reference #11).  Moreover, we have checked the internal consistency (reliability of the questionnaire), and it was found to be 0.82.  We have added the above statement on the modified manuscript from line # 144 to #152. |
| As we all know in our country, micronutrient deficiency during pregnancy are common; however, FCS has been validated against quantity of caloric intake, it has not been validated against adequacy of macronutrients or micronutrients so, why don’t you supplement with other dietary methods? | Dear Reviewer,  We would like to thank you for pointing out this issue. We are optimist the issues that you raised here are crucial in making the finding stronger.  Again, we have taken the forwarded comment as one limitation of the study and the following statement have been included from # 144 to #152.  However, the following were our assumption to use FCS in this study.  As you know it very well there are plenty of studies in Ethiopia those aimed to assess the dietary quality during the period of gestation. Those studies in Ethiopia including the study area t assessed diet diversity among pregnant women. These methods of dietary assessment do not give weight for different food items based on their nutritional value. However, FCS gives weight for each food items by quantifying the calorie intake.  Moreover, FCS gives weight for each food items which indicates the importance of each food group; not mention, consideration of diet diversity and food frequency over seven day period.  Therefore, FCS is a robust measure that considers other dietary methods like diet diversity.  To strengthen the aforementioned justification the following statement has been included from line # 144 to #152.  ‘Food consumption score is a composite variable that is constructed based on the following criteria: food frequency, diet diversity and relative nutritional value of each food item’ |
| To assess economic status, you mentioned the wealth index was used even if you did not mention how you conducted and how you made the analysis, wealth index is most commonly applied to rural communites since they do not know their exact economic source or monthly income and have agricultural products but your study setting was urban? so, why you conduct wealth index? | Dear Reviewer,  We have tried to explain how we conducted wealth index from line #156 to #158.  While conducting the wealth index we have used principal component analysis. There were 15 different variables that were considered for analysis.  As to the reason of using wealth index over income, wealth index is an important measure of someone’s living standard. Besides, it can help us to see unequal access to health services.  Moreover, it is difficult to exactly measure the income and expenditure of individuals to ascertain their economic status even in urban settings. |
| **Analysis:** the statistical analysis is not adequate Lack of necessary data How do you analyse attitude? | To assess the attitude of mothers towards the consumption variety of diets we used 4 questions. It was 5 item Likert scale questionnaire responses ranging from strongly agree to strongly disagree.  To assess the attitude of mothers towards the consumption variety of diets we used 4 questions. It was 4 item Likert scale questions with responses ranging from strongly agree to strongly disagree.  The following has been written in the modified manuscript from line # 158 to # 162.  ‘The attitude of the study participants towards the consumption diversified diet was measured using a 4 item Likert-scale questions, the response ranges from strongly disagree to strongly agree. It was considered favourable attitude when respondents score above the median. The internal consistency of the questionnaire was checked using Cronbach’s alpha (0.78). ’ |
| **Result:** rewrite the result systematically, and the tables need to be summarized Some of your variables of categories have a too small frequency e.g. maternal education which accounts less than 10% | Dear Reviewer,  Thank you for your constructive comments. In this section of the result unable to read and write, and can read and write have been aggregated into no formal education in the modified manuscript (Table 1) page.  However, in identifying associated factors with the outcome variable the expected count for the cells in these characteristics was >5. |
| **Discussion**: correct more than half is 51.2% more than half? | Dear Reviewer,  We have rephrased the adjective more than half as a little over half in the modified manuscript- line # 251. |
| Your discussion was not written coherent manner, and it does not keep the writing flow, you just once mentioned local and then abroad studies . | Dear Reviewer,  We have accepted your comments, the coherence has been fixed; by first writing the report from Bangladesh to Nigeria and finally writing the reports from Ethiopia. The change can be seen from the modified manuscript line # 253 to # 256. |
| And while you justify it was mentioned that the study provided greater evidence on dietary quality how? | Dear Reviewer,  The use of FCS provides greater evidence on dietary quality than diet diversity score. The use of FCS considers not only dietary diversity and food frequency but also the relative nutritional importance of different food groups.  Besides, unlike the use of dietary diversity score (DDS) which assess dietary over a 24 hours period; FCS provides evidence over 7-day period. |
| You utilized only FCS how do you measure dietary quality? | Dear Reviewer,  Thank you for raising the importance point. FCS considers the consumption different food groups over seven day period. Even though it has limitations in assessing the adequacy of macronutrients or macronutrients, it assess the relative importance different food groups.  Therefore, assessing different food groups over seven period will help us to see the quality of foods. |
| What measures did you take to manage recall bias? | Dear Reviewer,  Thank you for raising this important point, as we have tried to explain at #299. we have used photograph probes of the listed foods to minimize recall bias. |
| **Conclusion:** rewrite the conclusion that is out of your result | Dear Reviewer,  Thank you for your comments, now we have made some modifications. |
| **Reference**: there are too old reference better to use latest sources | Dear Reviewer,  We have accepted your comments, in the modified manuscript reference 6, 27 and 31 have been replaced with latest similar sources. |

**Reviewer 2:**

| **Reviewers report** | **Authors’ response** |
| --- | --- |
| Abstract |  |
| Background: Better to indicate the magnitude of the problem | Dear Reviewer, I would like to offer for your insightful comments. This time we have incorporated a sentence that indicates the magnitude of the problem. It can be found at line # 31 in the clear version of the manuscript. |
| Show the gaps that your study going to fulfill | Dear Reviewer,  The gaps we intended to fill has been indicated from line # 32 to # 34, which ‘to assess food consumption score and associated factors among pregnant women attending ante natal care services in health centers of Addis Ababa, Ethiopia.’ |
| Methods: |  |
| Line 33: incomplete sentence? | Dear Reviewer,  Thank you for pointing out the sentence, this time we have edited the sentence as ‘Overall, 999 pregnant women were selected for this study.’ |
| Line 33-35: Jargon words, rephrase it | Dear reviewer,  The word required in line number 33 was removed, and it has been rephrased as: ‘Overall, 999 pregnant women were selected for this study.’ Besides, the word recruit has been removed and replaced with a more familiar word- include. The change can be seen from line # 36-38. |
| Line 35: The duration of the study is very short only 30 days, so how this is considered for the analysis? | Dear reviewer,  The time duration indicated there is not the total duration of the study, but it indicates the duration the actual data collection period. |
| Indicate how to declare statistical significance of factors and measure of association used | Dear reviewer,  We have accepted your comment, and the following statement has been included from line # 43 to # 46.  ‘Crude and Adjusted Odds Ratio were used to assess the strength of the association. In the final model, p value < 0.05 was used to declare significance.’ |
| 1.3. Conclusion:  Better to restate your finding | Dear reviewer,  We have restated our finding saying ‘Besides, not skipping meal, having better educational status, wealth status and attitude were associated acceptable food consumption score.’  The added statement can be seen on the modified manuscript from line #56-#59 |
| Line 48-49: specify the areas of nutrition education to be provided | Dear reviewer,  Thank you for your comments, we have modified the manuscript based on your comments. The change can be seen on line # 58 on the modified manuscript. |
| Background |  |
| Show the burden or severity of the problem logically from global to local | Dear Reviewer,  We have accepted you comment and we have tried to show the burden of the problem from global to local.  The amended statement can be found from line # 78 to # 81. |
| Indicate any program or policy intervention taken previously, any success or failures  Show any gap that was answered by this study | Dear Reviewer,  Thank you for your comments, in the modified manuscript we have tried to show the actions that has been taken, and the existing gaps by citing two references- reference #17 and #18.  Moreover, this study has tried to show factors that was associated with food consumption pattern among pregnant women (line # 86 to # 89). |
| Indicate the contributions (theoretical or practical) of this study | Dear reviewer,  We have tried to explain the theoretical contribution of the study from line # 96 to # 100 in the modified manuscript. |
| Line 83-85: tell us how ordinal logistic regression makes your study unique from the others | Dear reviewer,  Different studies which were previously conducted among pregnant women in Ethiopia employed binary logistic regression to identify associated with FCS. In those studies the outcome variable was considered as binary (acceptable and unacceptable food consumption score). However, food consumption score categorized into three categories: poor, borderline and acceptable FCS. Therefore, ordinal logistic regression is more precise than binary logistic regression to identify associated factors (Reference #22). |
| 3.Methods |  |
| 3.1. study area:  show total number of reproductive age group women, and total number of pregnant women attending ANC | Dear Reviewer,  We have accepted your comment, the number of pregnant women who were coming for ANC service in the selected health centers have been included as follows from line # 111  ‘In these health centers, there were 2478 mothers who came for ANC services.’ |
| Indicate the food security status, and nutritional status of pregnant women in the study area | Dear Reviewer.  Thank you for you comments. In the amended manuscript we have included food security status of PSNP beneficiaries in the study area.  This can be accessed at line #107. |
| Include the climatic condition of the study area | Dear Reviewer,  Thank you for your comment, now we have included a statement that indicate the climate condition of the city. This can be accessed in the modified manuscript at line # 105. |
| 3.2. Sample size: |  |
| Sample size must be calculated for each objective and then the largest one must be considered | Dear Reviewer,  We would like to thank you for raising an important point. We have calculated the sample size for each specific objective, and we have taken the highest.  Therefore, we have included statements that explain how we estimated sample size for each specific objective and the largest was taken.  The added statement can be obtained in the modified manuscript from line # 114 to # 123. |
| Line 99: Why you considered COR? Is that recommended? | Dear Reviewer,  While calculating the sample size, we considered assumptions from previous studies. Likewise, in this study we considered expected odds ratio of attitude towards consumption of the consumption of a diversified diet on FCS in the absence of other factors.  Besides, while computing the sample size with the second objective proportion outcome (acceptable FCS) among the exposed and unexposed extracted from the previous study results COR.  Moreover, it usually recommended to use AOR if we were using the same variable. However, we have considered different variables to estimate the sample size, after which we have taken the variable (Attitude) that has given us the highest estimate. |
| Indicate population your inclusion criteria for your study | Dear reviewer,  Again, thank you for your constructive comment. We have now included a statement that indicate the inclusion criteria from line # 124 to # 132 in the modified manuscript. |
| The sampling techniques are not clear, better to use schematic presentation of sampling techniques | Dear reviewer,  We have used a multistage sampling technique. We have added the following statement to make the sampling technique more clearer: ‘ Out of the nine sub-cities in Addis Ababa, four sub cities were selected randomly (30%) In the selected sub cities there were 28 health centres.’  The above statement can be accessed in the modified manuscript from line # 124 to # 132. |
| 3.3. Data collection tools:  your data collection tool used for attitude is not clear? | To assess the attitude of mothers towards the consumption variety of diets we used 4 questions. It was 5 item Likert scale questionnaire responses ranging from strongly agree to strongly disagree.  To assess the attitude of mothers towards the consumption variety of diets we used 4 questions. It was 4 item Likert scale questions with responses ranging from strongly agree to strongly disagree.  The following has been written in the modified manuscript from line # 158 to # 162.  ‘The attitude of the study participants towards the consumption diversified diet was measured using a 4 item Likert-scale questions, the response ranges from strongly disagree to strongly agree. It was considered favourable attitude when respondents score above the median. The internal consistency of the questionnaire was checked using Cronbach’s alpha (0.78). ’ |
| did you checked the internal consistency of the tools? | Dear reviewer,  Yes, we have checked the internal consistency of the tools. To check the internal consistency of the tools we have used Cronbach’s alpha. Accordingly, the Cronbach’s alpha value for the outcome and the independent variable-attitude were 0.82 and 0.78, respectively. This can be accessed on line # 152 and line # 162 on the modified manuscript. |
| How did you measure attitude? | To assess the attitude of mothers towards the consumption variety of diets we used 4 questions. It was 5 item Likert scale questionnaire responses ranging from strongly agree to strongly disagree.  To assess the attitude of mothers towards the consumption variety of diets we used 4 questions. It was 4 item Likert scale questions with responses ranging from strongly agree to strongly disagree.  The following has been written in the modified manuscript from line # 158 to # 162.  ‘The attitude of the study participants towards the consumption diversified diet was measured using a 4 item Likert-scale questions, the response ranges from strongly disagree to strongly agree. It was considered favourable attitude when respondents score above the median. The internal consistency of the questionnaire was checked using Cronbach’s alpha (0.78). ’ |
| Line 127: better to provide reference used for wealth status | Dear reviewer,  Thank you for the comment, now we have cited our source to define wealth index. The provide reference is reference #23 in the modified manuscript. |
| Line 143: Illiterate, this is offensive word. | Dear Reviewer,  Thank you for pointing out this important point. In the modified manuscript the word ‘illiterate’ has been replaced with ‘unable to read and write’ |
| Line 145: Specify how did you ensured their confidentiality | Dear Reviewer,  To ensure confidentiality, we did not use a person identifier that exactly show the identity of the study participants. The completed and the filled data were only accessed by the investigators. The following statement has incorporated in the amended manuscript from line # 181-#182.  ‘ no person identifiers were used and the kobo account was password protected-only authorized user was able to access the data.’ |
| Results |  |
| Table 1: What was your bases to say small and large family size | Dear reviewer,  We have accepted you comment. The categories under the variable family, small and large family size have been amended as ≤4 and >4, respectively for small and large family size. This can be accessed in the modified manuscript on table 1 and table 6. |
| Table 2: 32.98% of your study participants were not attend ANC, but you conducted this study among pregnant women attending ANC services in your study area. so where did you get those not attending ANC | Dear Review,  That is exactly right, the study was conducted among pregnant women attending ANC service. However, this variable indicates if the pregnant woman had attended ANC service before this for the current pregnancy.  The question for this variable was:  ‘Have you ever had ante natal care visit other than today for this pregnancy?’  Therefore, 32.98% indicates pregnant women who had not attended earlier ANC services for the current pregnancy.  To make it clearer we have edited the variable as follows: ‘Earlier ANC visit for the current pregnancy’ Table 2 |
| 5. Discussion |  |
| Line 228: Better to specify the variable categories of wealth status and attitude which were statistically significant | Dear Reviewer,  Thank you for your comment. This time the categories of the variables which were significant have been written. The change can be seen in the modified manuscript from line # 264 to # 266. |
| Line 259-260: Tell us how could it be? | Dear reviewer,  Other previous studies that assessed predictors of food consumption score among pregnant women used binary logistic regression to identify independent predictors FCS. However, in our study we employed ordinal logistic regression which is better to predict associated factors. There are evidences that support this claim, reference #22. |

Reviewer 3:

| **Reviewers report** | **Authors’ response** |
| --- | --- |
| Line 29 “………to prevent these problems…….” Which problems are you talking about rewrite the statement or correct it. Again the background session lack the gap. | Dear Reviewer,  Thank you for your comments. Now on the background section the abstract has been rewritten showing the gaps.  The change can be seen from line #29. |
| Line131-132: “Multicollinearity was checked using Variance inflation factor (VIF<2)” why<2? What is your reference? This needs strong justification | Dear reviewer,  Variance inflation factor (VIF) was used to check for multicollinearity between independent variables. It is recommended the VIF to be near to 1 with a cut off point of 10. Thus, in our study the we did not find a VIF that was >2. |
| 132: “After checking all the assumptions of ordinal logistic regression” what are those assumptions explain them | Dear Reviewer,  The assumptions of logistic regression that we checked were:   1. Chi square assumption, there was no expected count in a single cell that was <5 2. Proportions odds assumption (Brant test of parallel regression assumption (p value = 0.665))   These were the assumption that we checked before fitting ordinal logistic regression. |
| Line 148: your sample size was 999; only 948 individuals participated in the study. How you missed them? | Dear Reviewer.  Of 999 sampled population 51 respondents were missed in this study. The reason for missing these respondents were as follows:   1. Of the collected data 37 were incomplete which were not appropriately uploaded to the kobo tool box. 2. 14 respondents drop out without answering all the questions despite relentless efforts of the data collectors and the supervisors. There reason for not completing the questionnaire were;    2.1. They were in hurry    2.2. Language barriers |
| What is the response rate? | Dear reviewer,  We have now written the response rate on the modified manuscript. This can be accessed on line # 184 on the modified manuscript. |
| Discussion session missed implication of the study | Dear reviewer,  The implication of the study is now included in the modified manuscript, it can be accessed from line # 306 to # 311. |
| Conclusion: What will be your recommendation for future research? | Dear Reviewer,  We would like to thank for your constructive comment. Now, recommendations for future researchers have been incorporated in the modified manuscript line # 323 to # 326. |
