# Supplementary material for: Food consumption score and predictors among pregnant women attending antenatal care services in health centers of Addis Ababa, Ethiopia: Using ordinal logistic regression model
Source: PLoS One. 2024 Jun 26;19(6):e0306169. doi: 10.1371/journal.pone.0306169 (PMC11207176; doi:10.1371/journal.pone.0306169)
Supplement: Attachment — Submitted filename: plos recomendation- coments.docx [file pone.0306169.s004.docx]

**Methodology**

You have used multistage sampling but you randomly selected why you have used 1.5 designs effect?

Number of health centers didn’t mentioned. please specify number of health centers.

Analysis

Where is your ordered outcome? To conduct ordinal logistic regression and just you said that I have conducted ordinal regression no command no assumption test, no ordinal outcomes seen.
