# Supplementary material for: Food consumption score and predictors among pregnant women attending antenatal care services in health centers of Addis Ababa, Ethiopia: Using ordinal logistic regression model
Source: PLoS One. 2024 Jun 26;19(6):e0306169. doi: 10.1371/journal.pone.0306169 (PMC11207176; doi:10.1371/journal.pone.0306169)
Supplement: Attachment — Submitted filename: Response to reviewers.docx [file pone.0306169.s005.docx]

**Date: August 29, 2023**

To: Plos One, Editorial Office

Subject: **Submitting a revised version of manuscript and point by point response**

Manuscript ID: PONE-D-23-02910R2

Title: Food consumption score and predictors among pregnant women attending antenatal care services in health centers of Addis Ababa, Ethiopia: using ordinal logistic regression model

We would like to thank the editor and the reviewers for sharing their view and experience on the manuscript. We have found the comments are very important, which will improve the quality of our manuscript. The point-by-point responses for each of the comments and the revised manuscript are provided in the attached documents.

Regards,

Authors

**Point by point response for reviewer four**

| **Reviewer’s request** | **Authors’ response** |
| --- | --- |
|  | Dear Reviewer,  We would thank you for your constructive comments. We believe your comments are crucial to our manuscript. Therefore, below are point by point response for the forwarded comments. |
| You have used multistage sampling but you randomly selected why you have used 1.5 designs effect? | Dear Reviewer,  We have decided to use design of 1.5 after postulating the homogeneity of the population of interest. And the large sample was obtained after using 1.5 design effect.  The finance issue was the other one. As funding was not acquired, collecting a data from very large sample would require mobilizing a huge resource including recruiting additional data collectors and supervisors. |
| Number of health centers didn’t mentioned. please specify number of health centers. | Dear Reviewer,  The number of health centers which were available during the data collection period is mentioned in line # 109, and the number of health centers from the randomly selected sub-cities were 28, and again nine health centers were selected from 28 health centers proportionally. This change can be seen from line # 128 to #133 in the modified manuscript.  ‘*Out of the ten nine sub-cities in Addis Ababa, four sub cities were selected randomly (30%): Nifas silk lafto sub-city, Kolfe keraniyo sub-city, Lideta sub-city and Akaki kality sub-city. In the selected sub- cities there were 28 health centres. First, nine health centres (one from Nifas silk lafto sub-city, two from Kolfe keraniyo sub-city, three from Lideta sub-city and three Akaki kality sub-city) were selected randomly using a lottery method.*’ |
| Analysis Where is your ordered outcome? To conduct ordinal logistic regression and just you said that I have conducted ordinal regression no command no assumption test, no ordinal outcomes seen. | Dear reviewer,  Our ordered outcome is food consumption score (FCS). Food consumption score (FCS) was categorized in to poor food consumption score, borderline food consumption score and acceptable food consumption score.  This can be seen in line # 158 in the modified manuscript.  Dear reviewer,  To run the ordinal logistic regression, as you have mentioned it, the first assumption is that there need to ordered outcome, which in our case was food consumption score categorized into three mutually exclusive categories: poor food consumption score, borderline food consumption score and acceptable food consumption score (line #158).  The other assumption was checked, which was non- existence of multicolinearity between independent variables- using variance inflation factor (VIF). This can be seen in line #170.  Finally, before running ordinal logistic regression we have checked proportion of odds assumption using Brant test, p value 0.66). This can be seen in line #170 in the modified manuscript. |

**Point by point response for reviewer Five**

| **Reviewer’s request** | **Authors’ response** |
| --- | --- |
| Dear Author! my general comments on your article were list below | Dear Reviewer,  As always, we have found your constructive comments helpful for the betterment of our manuscript. Based on your comments, below are point by point response for your constructive comments. |
| Between line #29 & #30 it is better if you add the statements that shows the presence of gap on the topic | Dear reviewer,  In the manuscript we have tried to address the existence of gap by pointing out the following statements in the abstract section of the modified manuscript.  ‘*Understanding the food consumption score can be used to prevent problems associated with poor dietary intake of pregnant mothers. In Ethiopia, the food consumption score ranges from 54% to 81.5%, which is far below the world food program recommendation.’* |
| In line # 33 & #34, why you prefer the word ‘required’… ‘Recruit’? it is better if you replace by the appropriate words | Dear Reviewer,  In the modified manuscript the word ‘recruit’ has been replaced with ‘selected’ when appropriate. This change can be seen in line#135 in the modified manuscript.  As to the word ‘required, in the modified manuscript the word required has been replaced with selected when appropriate. This change can be seen in line #123 in the modified manuscript. |
| In line # 33, #101 & #104, when you used ‘multistage sampling techniques’, did you decided or calculate the design effect? Why? | Dear Reviewer,  We have decided to use design of 1.5 after postulating the homogeneity of the population of interest. And the large sample was obtained after using 1.5 design effect.  The finance issue was the other one. As funding was not acquired, collecting a data from very large sample would require mobilizing a huge resource including recruiting additional data collectors and supervisors. |
| In line # 1 your topic of research & in line #86, food consumption patter & associated…’ please fix your title of the study. The two points are different | Dear Reviewer,  Thank you for pointing out this important point, we have accepted your comment the word ‘Pattern’ in line #86 has been replaced with score. This change can be seen in line # 86 in the modified manuscript. |
| In line # 91, and when you were responded for the 2nd reviewer question, you said “ nine sub city, four were selected first…, then nine health centers were again selected from 28 Health Centers” by what criteria you were exclude four out of total eleven sub cities of Addis Ababa? | Dear Reviewer,  As we have tried to explain it from line #128 to line # 133; we have selected four sub cities from the ten sub cities in Addis Ababa randomly. While selecting these sub cities randomly, we have used a lottery method.  This can be seen in line #133 in the modified manuscript. |
| In line # 35 & # 95, the study period and the actual data collection periods were mismatched. In my opinion, the study period duration has greater length than the actual data collection period duration. It is better if you adjust the period in a logical manner! | Dear Reviewer,  This was a typological error. As pointed in the methods section the actual data collection period was from June 07 to July 08, 2022. Therefore, the study period that was indicated in the methods section has been replaced with the actually data collection period. This change can be seen from line# 38 in the modified manuscript. |
| In line # 33, you got 999 sampled pregnant women. In line # 40 & # 148, and when you were responded to reviewer three, Of 999 sampled population you had 51 (37 + 14) missed respondents. So, when you subtract 51 from 999 your final respondents should be 948, not 949. You were mentioned language barrier as one reason for that fourteen (14) drop out respondents. In which language did you collected your data from the respondents? Why? Did you consider a diversity of languages with in the city? | Dear Reviewer,  We would like to apologize for the mistake, and we grateful for pointing out this error. Actually, the data was collected from 949 pregnant women. Therefore, there was 95% response rate. However, as we have tried to explain on previous response to reviewer three, there was 5% non-response rate; of this 36 was incomplete which were not appropriately uploaded to the kobo tool box.  Dear Reviewer,  The data was collected after translating the English version of the questionnaire to Amharic. Addis Ababa is a diverse city and different languages are spoken, officially Amharic is spoken in the city. However, some of the respondents were not able to speak Amharic. |
| Please make the numbers of decimal similar (i.e. either one or two numbers after decimal for all narratives). As an example in line #42, #161 and #181. | Dear Reviewer,  We have accepted your comments, and now there are two numbers after decimal. These changes can be seen through out the modified manuscript. For example line #224, line #225, line #258, line #260 and Table 6. |
| In line # 113, what were your criteria when selected your data collectors and supervisors? Why? | Dear Reviewer,  While selecting our data collectors and supervisors we have had two basic criteria, previous experience of working as a data collector or supervisor in a similar setting, and the other was educational background of the data collectors and the supervisors.  As we have tried to explain it on the modified manuscript from line #139 to #140, the data collectors were B.Sc. Nurses and the supervisors were public health officers.  The reasons for us to select those data collectors and supervisors were:   1. We used a computer-based data collection tool, kobo tool box which requires computer knowledge and previous experience to it or similar software. 2. As the study was institution based, in randomly selected health centers, it would be better to have someone who had experience working in such kind of environment. |
| Regarding independent predictor condition of wealth status, in line # 44 ‘ being in the poorest wealth status’ Check your analysis or reference category for this variable , in line # 269 to #270 ‘not being from the poorest household’ were independent predictor of acceptable food consumption score. Which one is correct? The former or late line? | Dear Reviewer,  Thank you for pointing out this important issue. As we have tried to discuss it, being from the poorest household decreases food consumption score by 49% (AOR=0.52, 95% CI: 0.34-0.78).  Therefore, the later is correct and the former at line # 53 has been modified.  The change can be seen in line # 53 in the modified manuscript.  ‘*Not being from in the poorest wealth status’* |
| Did you have a model or image you showed for the participants to respond the like rt scale questions? Please explain or include the model or image into the document | Dear reviewer,  Yes, we have used photograph images to recite memory of the study participants (line #306). Those photographs were used to recite the memory of the study participants.  The images of the food items were shown to the participants by the data collectors during the interview to recite the memory of the study participants. |
| What is your reference to categorize attitude into favorable and unfavorable rather than positive and negative attitude? | Dear Reviewer,  In the revised manuscript attitude has been categorized into positive and negative attitude.  The changes can be seen in line #54, line #121, line #163, line #197, line #248, line #273, and line #326. And this modification has also been applied in table 1 and table 6.  However, a study from Easten Ethiopia has used the former classification, favorable and unfavorable attitude (Reference 12, in this manuscript). |
| Include in your recommendation section for the young or future researchers who need to research on the area! | Dear reviewer,  Recommendation for young or future researchers have been included in the modified manuscript. This can be seen from line # 329 to line #332.  ‘*Future researches are encouraged to investigate the validity of FCS to assess adequacy of macronutrients and micronutrients among pregnant women. Moreover, future studies triangulated with qualitative research that investigate behavioural factors such as food taboos and norms that influence FCS among pregnant women are also encouraged.’* |
| Do you say something about your level of acceptable food consumption score (51.2%) when compared with national acceptable food consumption score (54%)? | Dear Reviewer,  In the introduction section we have written that food consumption score in Ethiopia ranges from 54% to 81.5%. This point was brought to imply FCS in Ethiopia is far below world food program recommendation. |
