# Supplementary material for: Food consumption score and predictors among pregnant women attending antenatal care services in health centers of Addis Ababa, Ethiopia: Using ordinal logistic regression model
Source: PLoS One. 2024 Jun 26;19(6):e0306169. doi: 10.1371/journal.pone.0306169 (PMC11207176; doi:10.1371/journal.pone.0306169)
Supplement: Attachment — Submitted filename: Comment on o FSC.docx [file pone.0306169.s006.docx]

**Comments to the Author:**
**Title**: Food consumption score and predictors among pregnant women attending antenatal care
services in health centers of Addis Ababa, Ethiopia: using ordinal logistic regression model. The manuscript is well written, and it gives good insights on predictors of the recommended acceptable food consumption score and when these factors are given good attention it will improve inadequate dietary diversity. However, I forwarded the following minor comments to improve the manuscript.
**General comment**

Has language and some editorial problems. Especially the results &discussion part.

**Abstract**

- Background: the first sentence is too long and makes it difficult to understand the message. Therefore, make it two sentences.
- Result: “**not being from** the poorest wealth status (AOR=0.52, 95% CI: 0.34-0.78)”. It is good to put the result as it is rather than interpret it.
- Conclusions: line 58; what attitude is associated with acceptable food consumption
   score?

**Methods**

Data collection tools and measurement

Line 137 “…questionnaire that comprises
 socio-demographic data, dietary habits, **attitude obstetric history**…” What is attitude obstetrics history?

**Result**

Table 1: Husband's educational status: Husbands who attended a primary and above educational status can’t read and write? Please re-write as that of the mother's educational status classification.

Table 2: What is the recommended birth interval and non-recommended birth interval? It is vague.

Again you classified by trimester and then by having early ANC. What is the difference between the first trimester and “early ANC”? What is the input of these classifications for you?

**Factors associated with food consumption score**

The classification of maternal educational status is not as that of Table 1. Which is confusing. Either classify as 1. Can’t read and write 2. Can read and write. Or you can use the mother educational status classification on your table and regress it again.

**Discussion**

On line 219 “The study period
 **could** explain…” Are sure that? Have you controlled everything??? I think the language should have to be corrected.

On line 223; “…physiological …” what is the unique physiological difference between the pregnant women of your study area and the other conducted studies?

On lines 225 -226; “…However, the FCS outcome in our finding is much higher than a study conducted in the Federal University of Agriculture, Abeokuta (FUNAAB)-19.4%.” So, what is your possible explanation for this discrepancy?

On lines 228-229; “…our study has shown **maternal nutritional education**…were independent predictors of FCS. Is it maternal education/maternal educational status that was an independent predictor?

**Conclusion**

Line 274-275**; “…**provide intensive nutritional education prioritizing pregnant women with lower socio-economic status and wealth status…” On this particular recommendation, how that intensive nutritional education improve low socio-economic status? / What do you recommend for?
