# Supplementary material for: Food consumption score and predictors among pregnant women attending antenatal care services in health centers of Addis Ababa, Ethiopia: Using ordinal logistic regression model
Source: PLoS One. 2024 Jun 26;19(6):e0306169. doi: 10.1371/journal.pone.0306169 (PMC11207176; doi:10.1371/journal.pone.0306169)
Supplement: Attachment — Submitted filename: Response to reviewers.docx [file pone.0306169.s007.docx]

**Date: November 20, 2023**

To: Plos One, Editorial Office

Subject: **Submitting a revised version of manuscript and point by point response**

Manuscript ID: PONE-D-23-02910R2

Title: Food consumption score and predictors among pregnant women attending antenatal care services in health centers of Addis Ababa, Ethiopia: using ordinal logistic regression model

We would like to appreciate the editor and the reviewer for sharing their insightful remarks and experience on the betterment of the manuscript. As usual, we have found these remarks crucial in augmenting the quality of our manuscript. Therefore, the following are point-by-point responses for the raised comments and remarks. We have also submitted the documents (revised manuscript and the revised manuscript with track changes) edited based on the editor’s and reviewer’s comments.

Regards,

Authors

**Point by point response to the Academic editor**

| **Academic Editor’s request** | **Authors’ response** |
| --- | --- |
|  | **Dear Editor,**  We are grateful for your usual constructive comments, below are point by point response to the raised questions and comments. |
| Assessment of variables, results, and recommendations are not clear. | **Dear Editor,**  To make the mentioned sections clearer we have made a language and punctuation editions. |
| Please make the entire manuscript legible | **Dear Editor,**  We do appreciate your remarks, this time we have made language and punctuation editions. |

**Point by point response to the reviewer,**

| **Reviewer’s request** | **Authors’ response** |
| --- | --- |
| **General comment:** Has language and some editorial problems. Especially the results &discussion part | **Dear Reviewer**,  We are thankful for the constructive remarks and comments. This time, we have made language and punctuation editions. |
| **Abstract**  Background: the first sentence is too long and makes it difficult to understand the message. Therefore, make it two sentences. | **Dear reviewer,**  We have accepted your comment, it has been amended as follows:  **“***Poor maternal nutrition during pregnancy creates a stressful environment that can lead to long-term effects on tissue development. ”* |
| **Result:** “not being from the poorest wealth status (AOR=0.52, 95% CI: 0.34-0.78)”. It is good to put the result as it is rather than interpret it. | **Dear reviewer,**  We have modified it as ‘poorest wealth status’ |
| **Conclusions:** line 58; what attitude is associated with acceptable food consumption  score? | **Dear reviewer,**  We have revised it and tried to make it clearer as follows:  “Attitude towards consumption of a diversified diet” |
| **Methods**  Data collection tools and measurement Line 137 “…questionnaire that comprises socio-demographic data, dietary habits, attitude obstetric history…” What is attitude obstetrics history? | **Dear reviewer,**  It was a typological error, but now we have separated the two words with comma, now it has been clearly stated as follows:  *“…dietary habits, attitude towards consumption of a diversified diet, obstetric history …”* |
| **Result** |  |
| Table 1: Husband's educational status: Husbands who attended a primary and above educational status can’t read and write? Please re-write as that of the mother's educational status classification**.** | **Dear Reviewer**,  We are grateful for your insightful remark, accordingly, it has been classified as 1. Can’t read and write 2. Can read and write **.`** |
| Table 2: What is the recommended birth interval and non-recommended birth interval? It is vague. | **Dear Reviewer,**  According to the WHO, birth interval of less than 24, which the time from last birth to conception of the current pregnancy is not a recommended interpregnancy birth interval.  Therefore, the category was made based on the WHO recommendation as recommended birth interval when it is more than 24 months, otherwise it was categorized as non-recommended birth interval.  To make it clearer, we have included the definition of the term in the methods section.  **“***Finally, birth interval was categorized as recommended birth interval when interpregnancy interval was more 24 months otherwise it was categorized as not recommended birth interval”* |
| Again, you classified by trimester and then by having early ANC. What is the difference between the first trimester and “early ANC”? What is the input of these classifications for you? | **Dear Reviewer,**  Thank you for raising this important issue. Trimester, in the study indicates the gestational age during the data collection period. Accordingly, mothers who were less than 14 complete weeks of gestational age were categorized as first trimester, between 14-27 complete weeks of gestational age as second trimester, and 28 weeks of gestation age until delivery as third trimester.  “*The trimesters were defined as first trimester (less than 14 weeks), second trimester (14–27 weeks) and third trimester (28 weeks until delivery).”*  With regard to ANC, we have asked the pregnant mother if they had ANC follow-up ***earlier*** to the current ANC follow-up. Therefore, these mothers who had at least one ANC follow up before the current ANC was regarded as having ***earlier*** ANC. |
| Factors associated with food consumption score:  The classification of maternal educational status is not as that of Table 1. Which is confusing. Either classify as 1. Can’t read and write 2. Can read and write. Or you can use the mother educational status classification on your table and regress it again. | Dear Reviewer,  We are grateful for your insightful remark, accordingly, it has been classified as 1. Can’t read and write 2. Can read and write |
| **Discussion**  On line 219 “The study period  **could** explain…” Are sure that? Have you controlled everything??? I think the language should have to be corrected. | Dear Reviewer,  We have made modification in the language; it has been modified as follows:  ‘*Furthermore, this disparity may be explained by physiological and cultural factors that existed during the gestational period.’* |
| On line 223; “…physiological …” what is the unique physiological difference between the pregnant women of your study area and the other conducted studies? | Dear Reviewer,  Thank you for your insightful remark, that is right, there is little physiological difference between pregnant women living in different study areas. However, in this section we have discussed our findings with different studies which had a similar study group and/or similar outcome measurement. For example, our finding was discussed against the study from Nigeria (reference 27) which was conducted among traders.  Thus, the point is trying to explain the difference between the finding from Nigeraia and the current finding. |
| On lines 225 -226; “…However, the FCS outcome in our finding is much higher than a study conducted in the Federal University of Agriculture, Abeokuta (FUNAAB)-19.4%.” So, what is your possible explanation for this discrepancy? | Dear Reviewer,  The possible explanation for this discrepancy is:  ‘*This disparity may have been attributed to Ethiopia's recently implemented food and nutrition policy and strategy, which aims to increase nutrition literacy.’* |
| On lines 228-229; “…our study has shown **maternal nutritional education**…were independent predictors of FCS. Is it maternal education/maternal educational status that was an independent predictor? | Dear reviewer,  Thank you for your remark, we have made modification as ***maternal educational status***. |
| **Conclusion**  Line 274-275**; “…**provide intensive nutritional education prioritizing pregnant women with lower socio-economic status and wealth status…” On this particular recommendation, how that intensive nutritional education improve low socio-economic status? / What do you recommend for? | Dear reviewer,  Our recommendation is targeting pregnant woman who had lower odds of having acceptable food consumption score. Therefore, this recommendation targets pregnant women with low socioeconomic (poor wealth status-according to the WFP wealth status is a proxy indicator of household economic status) and lower educational status.  As it has been seen from different studies, it is not only the accessibility of foods that affects mothers’ food consumption score, but also their nutrition knowledge. Moreover, studies have revealed that mothers with good nutritional knowledge were able to engage in income generation activities.  Therefore, increased nutritional knowledge will increase mothers FCS. |
