# Supplementary material for: Food consumption score and predictors among pregnant women attending antenatal care services in health centers of Addis Ababa, Ethiopia: Using ordinal logistic regression model
Source: PLoS One. 2024 Jun 26;19(6):e0306169. doi: 10.1371/journal.pone.0306169 (PMC11207176; doi:10.1371/journal.pone.0306169)
Supplement: Attachment — Submitted filename: Response to reviewers.docx [file pone.0306169.s008.docx]

**Date: January4, 2024**

To: Plos One, Editorial Office

Subject: **Submitting a revised version of manuscript and point by point response**

Manuscript ID: PONE-D-23-02910R3

Title: Food consumption score and predictors among pregnant women attending antenatal care services in health centers of Addis Ababa, Ethiopia: using ordinal logistic regression model

We would like to appreciate the reviewers for sharing their insightful remarks and experience on the betterment of the manuscript. As usual, we have found these remarks crucial in augmenting the quality of our manuscript. Therefore, the following are point-by-point responses for the raised comments and remarks. We have also submitted the documents (revised manuscript and the revised manuscript with track changes) edited based on the editor’s and reviewer’s comments.

Regards,

Authors

**Point by point response to the reviewer,**

| **Reviewer’s request** | **Authors’ response** |
| --- | --- |
| The manuscript is well improved. But I have some critical recommandations and comments still on the next points. | Dear Reviewer,  As always, we would like to extend our gratitude for your insightful remarks. We have tried to answer and resolve the raised remarks based on your suggestions and comments.  We would also like to assure you that the responses we incorporated previously, and now are based on scientific evidences-either published articles or books. |
| On Table 2. ANC classification pat. It is still vegue for your readers. As I understand from your explanation It is better if you say “History of early ANC vist for the current pregnancy”. | Dear Reviewer,  Thank you for the suggested remark. We have accepted your comment, and it has been modified accordingly.  On the modified manuscript we have edited Table 2 as follows (Page 12):  “***History of early ANC visit for the current pregnancy***”. As you have suggested it, we have replaced ‘**E*arlier*** ANC visit for the current pregnancy’ with ‘***History of early*** ANC visit for the current pregnancy**’** to make the point clearer for our readers. |
| 1. On line 223; “…physiological …” what is the unique physiological difference between the pregnant women of your study area and the other conducted studies? Even though you can state some scientific explanation, still you persist on “physiological difference”. As far as I know there is no physiological difference between the Addis Ababa’s pregnant women and Nigerian or some else in the world. Because they are all human beings and we are using same standard books like Wiliams, Gabe…. Therefore, please make it scientific rather than guessing, | Dear Reviewer,  We have accepted your comment, this time we have made edits based on your constructive comment. Therefore, we have removed physiological difference. And it has been rephrased as follows:  *‘In Ethiopia, pregnant women avoid foods due to cultural and religious reasons, and this might explain the discrepancy between the current and the study from Nigeria where religion has lesser influence over their food choice.’*  *https://www.ncbi.nlm.nih.gov/pmc/articles/PMC8483102/*  The change can be seen from line# 269-271 in the edited manuscript.  **P.S.**  Dear reviewer, again, as you have clearly stated it, there is no physiological difference between pregnant women in Ethiopia and other parts of the world. In the previous comment, what we were trying to state was the difference in the study participates. Our study participants comprise only pregnant women while the other study was conducted from different population group-Market women- and this was what we have tried to explain previously. |
| 2. On lines 225 -226; “…However, the FCS  outcome in our finding is much higher than a study conducted in the Federal University of Agriculture, Abeokuta (FUNAAB)-19.4%.”  So, what is your possible explanation for this discrepancy?  Your response was: The possible explanation for this discrepancy is: ‘This disparity may have been attributed to Ethiopia’s recently implemented food and nutrition policy and strategy, which aims to increase nutrition literacy.  Of course the policy may play some role but,  The next question is are you sure that the Nigerian’s have no food and nutrition policy and strategy? They have endorsed before Ethiopia on 2001.  Read on: NATIONAL POLICY (nationalplanning.gov.ng)  So, it is good to see for another possible explanation. | Dear Reviewer,  Thank you for your insightful remark, and suggestion. We have gone through national policy on food and nutrition that was developed by the national planning committee of Nigeria in 2001. In addition to the time gap between the two policies, we have seen some differences in the objectives of the two policies: videlicet, the Ethiopian policy incorporated an objective that aimed at improving Nutrition Literacy of all Ethiopians. Therefore, this might have brought a positive change on food consumption of pregnant women residing in Ethiopia.  Moreover, after your critical remark we have seen the details of the study conducted in Nigeria- [(PDF) Street food consumption score and nutritional status of staff of federal university of agriculture, Abeokuta, Ogun state (researchgate.net)](https://www.researchgate.net/publication/345977303_Street_food_consumption_score_and_nutritional_status_of_staff_of_federal_university_of_agriculture_Abeokuta_Ogun_state). In this study, 49.7% of the study were male, and the remaining were non pregnant women staffs working at the university. Therefore, in the modified manuscript we have made edits by removing it.  This change can be seen from line # 272 to # 276 in the manuscript with track changes.  Again, we would like to reassure you that based on you suggestion we have critically reviewed the policies enacted by both African countries.  **Objectives of the National Policy on Food and Nutrition in Nigeria:**   1. *To improve food security at the household and aggregate levels to guarantee that families have access to adequate (both quantity and quality) and safe food to meet nutritional requirements for a healthy and active life;* 2. *To enhance care-giving capacity within households with respect to child feeding and child care practices, as well as addressing the care and well-being of mothers;* 3. *To improve the provision of human services, such as health care, environmental sanitation, education and community development;* 4. *To improve capacity within the country to address food and nutrition problems; and* 5. *To raise understanding of the problems of malnutrition in Nigeria at all levels of society, especially with respect to its causes and possible solutions*   **Objectives of Food and Nutrition Policy of Ethiopia:**   1. *Ensure the availability and accessibility of adequate food to all Ethiopians at all times.* 2. *Improve accessibility, and quality of nutrition and nutrition smart health services at all stages of the life span in an equitable manner* 3. *Improve consumption and utilization of a diversified and nutritious diet that ensures a citizen’s optimal heath throughout their life cycle.* 4. *Improve the safety and quality of food throughout the value chain.* 5. *Reduce food and nutrient losses along the value chain.* 6. *Improve food and nutrition emergency risk management, preparedness and resilience systems.* 7. *Improve food and nutrition literacy of all Ethiopians.* |
| **Recommendation**  3. Page 21 # line 333-337. Please revise the language and put your recommendation based only on your findings | Dear Reviewer,  Thank you for your insightful remark, based on your remark we have made a language edition on the recommendation. Besides, the recommendations are now solely emanated from the findings of our study.  The edit can be seen from line # 329 to line #399.  ***“****Therefore, it is important to give a special attention to pregnant mothers with low socioeconomic status, and mothers who skip meals in order to enhance their food variety score and improve their nutritional intake.”* |
