# Supplementary material for: Food consumption score and predictors among pregnant women attending antenatal care services in health centers of Addis Ababa, Ethiopia: Using ordinal logistic regression model
Source: PLoS One. 2024 Jun 26;19(6):e0306169. doi: 10.1371/journal.pone.0306169 (PMC11207176; doi:10.1371/journal.pone.0306169)
Supplement: Attachment — Submitted filename: Response to reviewers.docx [file pone.0306169.s009.docx]

**Date: March 05, 2024**

To: Plos One, Editorial Office

Subject: **Submitting a revised version of manuscript and point by point response**

Manuscript ID: PONE-D-23-02910R4

Title: Food consumption score and predictors among pregnant women attending antenatal care services in health centers of Addis Ababa, Ethiopia: using ordinal logistic regression model

We would like to appreciate the reviewers for sharing their insightful remarks and experience on the betterment of the manuscript. As usual, we have found these remarks crucial in augmenting the quality of our manuscript. Therefore, the following are point-by-point responses for the raised comments and remarks. We have also submitted the documents (revised manuscript and the revised manuscript with track changes) edited based on the editor’s and reviewer’s comments.

Regards,

Authors

**Point by point response to the reviewer,**

| **Reviewer’s request** | **Authors’ response** |
| --- | --- |
| Dear Author!, your manuscript was improved starting from Abstract to Reference. However, I need clarification for some points. | Dear Reviewer,  We are grateful for your constructive comments, we would like to assure you that your insightful remarks are enlightening not only on the betterment of the current manuscript, but on our future endeavors of publishing our research works on prestigious journals like Plos one. |
| 1. on Table 1, you classified marital status into currently married(919), and currently unmarried(30). Currently unmarried may include single, divorced, widowed etc. When I read the husbands educational status, you were included those currently unmarried numbers as well. How it possible? | Dear Reviewer,  As you have clearly stated it, small proportion of the study participants were unmarried (3.2%). Of those, 9 (0.9%), 7 (0.7%) and 14 (1.6 %) of these unmarried mothers were single, widows and divorced, respectively.  From those single mothers, we have asked them about the educational status of the father of the unborn child. Here, it is noteworthy that these single mothers had partners who were not officially married, at the data collection these mothers were not living with their partners (father of the unborn child) nor they were married.  In similar vein, divorced mothers were asked this same question, the educational status of the father. We ponder there were some incidents where married couples might be divorced even after being pregnant. This has been seen in urban areas of Ethiopia.  At to the widows, most of the widow mothers (6) were in the third trimester, only one was in the second trimester. As we go through the notes that we collected from the reports of our data collectors and the supervisors, the study participants (pregnant mothers) who were widows have told them that the fathers of the unborn children were died at most two weeks prior to the data collection period.  Therefore, even though, the fathers of the unborn children (single and divorced mothers) were not around, in urban areas fathers have close intimacy with mothers in supporting mothers during their pregnancy and raising children after they are born. |
| 2. In line#269, better if you write the percentage of acceptable FCS in the two studies done in Ethiopia | Dear Reviewer,  Thank you for your remark, we have included the percentage of acceptable FCS in the reviewed manuscript. The change can be seen on line #269 and # 270 in the modified manuscript. |
